# aEEG in the first 3 days after extremely preterm delivery relates to neurodevelopmental outcomes

**DOI:** 10.1038/s41372-024-01945-z

**Published:** 2024-03-29

**Authors:** Roberta Pineda, Zachary Vesoulis, Nathalie El Ters, Amit Mathur

**Affiliations:** 1 Chan Division of Occupational Science and Occupational Therapy, University of Southern California, Los Angeles, CA USA; 2 Keck School of Medicine, Department of Pediatrics, Los Angeles, CA USA; 3 Gehr Family Center for Health Systems Science and Innovation, University of Southern California, Los Angeles, CA USA; 4 Program in Occupational Therapy, Washington University St. Louis, St. Louis, MO USA; 5 Department of Pediatrics, Washington University School of Medicine, St. Louis, MO USA; 6 Department of Pediatrics, St. Louis University, St. Louis, MO USA

**Keywords:** Health care, Risk factors, Outcomes research

## Abstract

**Objectives:**

Investigate relationships between aEEG in the first 72 h in extremely preterm infants with 1) infant, medical, and environmental factors, and 2) infant feeding and neurobehavioral outcomes at term and school-age.

**Methods:**

Sixty-four preterm infants (≤28 weeks gestation) were enrolled within the first 24-hours of life and had two-channel aEEG until 72 h of life. Standardized neurobehavioral and feeding assessments were conducted at term, and parent-reported outcomes were documented at 5–7 years.

**Results:**

Lower aEEG Burdjalov scores (adjusted for gestational age) were related to vaginal delivery (*p* = 0.04), cerebral injury (*p* = 0.01), Black race (*p* < 0.01) and having unmarried parents (*p* = 0.02). Lower Burdjalov scores related to less NICU Network Neurobehavioral Scale arousal (*p* = 0.002) at term and poorer BRIEF global executive function (*p* = 0.004), inhibition (*p* = 0.007), working memory (*p* = 0.02), material organization (*p* = 0.0008), metacognition (*p* = 0.01), and behavioral regulation (*p* = 0.02) at 5–7 years. We did not observe relationships of early aEEG to feeding outcomes or sensory processing measures.

**Conclusion:**

Early aEEG within the first 72 h of life was related to medical and sociodemographic factors as well as cognitive outcome at 5–7 years.

## Introduction

Extremely low birth weight infants are at high risk for cerebral injury and neurodevelopmental impairment [1]. However, many infants born at early gestational ages go on to have positive developmental trajectories with minimal or no impairment [2]. Use of early neurobehavioral testing, cranial ultrasound, and MRI can aid in early identification of infants at high risk, but these measures are imperfect and have known issues with poor positive predictive value [3–6]. Further confusing providers, some infants without evidence of injury or impairment demonstrate developmental problems later, while others with moderate or severe injury develop without impairments. Alternative sources of predictive information are of great value to providers and parents to understand brain health and risk of adverse outcomes.

Distinct from static radiographic measures, electrocortical activity provides functional information about the brain. Electroencephalography (EEG) has historically been used to measure electrocortical activity in infants, children, and adults and has been demonstrated to have good prognostic accuracy [7]. However, EEG requires multiple leads to be applied to the scalp, long periods of monitoring, and expert interpretation by a neurophysiologist. An alternative approach is amplitude integrated encephalography (aEEG), which requires 5 leads (2 central, 2 parietal, 1 ground), can be done at the infant bedside without the need for extensive infrastructure, and interpretation that does not rely on an expert neurophysiologist. Although the primary use of aEEG is for real-time assessment of brain activity and the identification of suspected seizures, infants also display maturation-dependent patterns of activity [8, 9], which can be of particular importance (and potentially an indicator of injury) if the pattern does not match the postmenstrual age (PMA) at the time of aEEG recording.

Formal scoring systems have been developed to determine cerebral maturation from aEEG tracings [10, 11]. The Burdjalov score is commonly used in the United States [10]. Lower Burdjalov cerebral maturation scores are associated with earlier PMA. As the infant matures, the baseline variability rises, bandwidth decreases, and cyclicity becomes regular so that by the time infants reach term equivalent age, scores are significantly higher [9]. By comparing the actual PMA to the estimated PMA by aEEG, these scores can also provide valuable information related to cerebral health and well-being.

Variance in the total Burdjalov cerebral maturation score has been related to medical factors in the NICU such as higher illness severity at birth, vaginal delivery, sepsis, death, and longer periods of ventilatory support [12, 13]. In addition to relationships with medical severity, the Burdjalov cerebral maturation score at 30 weeks PMA has also been related to motor outcome at term equivalent age [12]. Finally, the Burdjalov cerebral maturation score at term equivalent age (38-42 weeks PMA) in formerly extremely preterm infants has been related to brain injury and motor and cognitive outcomes at 2-3 years of age [14, 15].

Studies of early aEEG, performed in the first 72 h after birth, have also demonstrated some predictive value. One component of the total Burdjalov cerebral maturation score, cyclicity, has been shown to be related to cerebral injury if not present within the first 24 h of birth in full-term infants [16]. When comparing two classification systems: Burdjalov versus Hellstrom-Westas, both scoring systems’ determination of cycling were associated with survival, and Hellstrom-Westas scores were also associated with Bayley motor and cognition scores at 24 months [17]. In other studies, Burdjalov cerebral maturation scores in the first 72 h after preterm birth have been shown to be related to MRI and outcomes in the first two years of life [18–20], and aEEG measures within the first 2 weeks of life have been related to outcomes at age 3 years [21]. Studies investigating relationships of early aEEG and EEG with school age outcomes are limited. In these studies, electrocortical activity patterns were related to cognitive outcome, adaptive behavior, and executive function that extended until 10–12 years [22, 23]. More recently, machine learning-based models have been used to demonstrate that aEEG in the first days after birth in extremely preterm infants predict childhood outcomes [24]. While there is limited knowledge about how early aEEG relates to school age outcomes, we are not aware of any studies that have investigated early aEEG related to feeding outcomes. Therefore, this study aimed to investigate relationships between aEEG in the first 72 h in extremely preterm infants with 1) infant, medical, and environmental factors, and 2) infant feeding and neurobehavioral outcomes at term and school-age.

## Methods

### Study cohort and procedures

The study protocol was reviewed and approved by the Washington University Human Research Protection Office, with a ceded review at the University of Southern California, and written informed consent was obtained by the parents prior to the start of any study procedures. Infants were enrolled in a prospective aEEG monitoring and MRI imaging study (aimed at understanding changes in the brain across hospitalization) at a single center, St. Louis Children’s Hospital, an 85-bed level IV referral NICU. Because of the use of this convenience sample from an overarching study, no power analysis was used to determine sample size. Inclusion criteria were birth at ≤28 weeks estimated gestational age (EGA), no antenatal diagnosis of congenital or chromosomal anomalies, and enrollment within the first 24 h of birth. Consecutive NICU admissions of infants born from 2014 to 2016 were recruited.

All enrolled infants underwent bedside two-channel aEEG monitoring as soon as was practical after enrollment through 72 h of life. Standardized neurobehavioral and feeding assessments were conducted between 34 weeks PMA and 42 weeks PMA. At 5–7 years of age, parent-report measures of developmental outcomes were completed. Evaluators of neurobehavior and feeding at term, as well as the parents completing questionnaires at 5–7 years, were blinded from aEEG measures taken within the first 3 days of life.

### Maternal and infant factors

Maternal factors that were collected included dichotomous categorical variables of marital status (married or single), prenatal smoking, prenatal use of alcohol, prenatal illicit drug use, use of prenatal steroids, mode of delivery (vaginal or Caesarean), maternal medications (magnesium, indomethacin), maternal anesthesia (none, general, or spinal), use of antibiotics during labor, and maternal education (some college or no college). Most variables were collected from the electronic medical record, with additional information collected from a questionnaire completed prior to NICU discharge (prenatal smoking, alcohol use, maternal education, and illicit drugs).

Infant factors that were collected included the following continuous variables: EGA, birthweight, occipital-frontal circumference at birth, Apgar scores at 1 and 5 min, Clinical Risk Index for Babies II (CRIB-II) score, cord gases immediately after birth, total oxygen hours, length of stay, as well as the number of days on total parenteral nutrition (TPN), high frequency oscillatory ventilation (HFOV), endotracheal intubation, continuous positive airway pressure (CPAP), nasal cannula, and breast milk. Categorical variables included race (Black or not Black), insurance type (public or private), infant sex (male or female), cerebral injury (defined by cranial ultrasound and MRI and dichotomized into ‘moderate to severe injury’ – cerebellar hemorrhage, grades 3-4 intraventricular hemorrhage and/or cystic periventricular leukomalacia or ‘no to mild injury’– the absence of the aforementioned injuries throughout the NICU stay), as well as presence or absence of patent ductus arteriosus - requiring Indomethacin or surgical ligation [25], necrotizing enterocolitis (NEC) – Bell’s stage IIb or greater [26], retinopathy of prematurity (ROP)-requiring surgery or Bevacizumab, and whether the infant expired during their NICU hospitalization.

Environmental factors collected included the NICU room type (private room or open bay), concurrent (during the aEEG tracing) endotracheal intubation, and concurrent use of sedatives.

### aEEG recording

aEEG was acquired using the BrainZ BRM3 (Natus Medical Incorporated, Pleasanton, CA), a two-channel bedside aEEG monitor that displays raw and amplitude-compressed recordings for each cerebral hemisphere. Hydrogel electrodes (Natus Medical Incorporated, Pleasanton, CA) were placed on the infant’s scalp in the central (C3-C4) and parietal (P3-P4) locations. The aEEG recording was started as soon after consent as was practical and was continued until 72 h after birth.

aEEG recordings were made only in an observational context as part of the research project and were not used to direct patient care. Interpretation and analyses of tracings occurred after hospital discharge. Recordings were retrieved from the monitors and reviewed using manufacturer supplied software (AnalyZe, Natus Medical Incorporated, Pleasanton, CA). Recordings with persistent high impedance, defined as impedance >20 kW were excluded.

### aEEG Burdjalov scoring

Using a 4-h long, artifact-free segment near the midpoint of the recording conducted in the first 72 h, recordings were evaluated for background pattern, onset and appearance of cyclicity, and lower amplitude border and bandwidth, which were used to derive a composite Burdjalov score [10]. The 4-hour long midpoint was selected to standardize the time period used for aEEG analysis. The Burdjalov score ranges from 0–13 and increases with higher PMA. Cyclicity, defined as at least one cycle of sinusoidal variation in minimum amplitude alternating between narrow and broad bandwidths, was also recorded as present or absent for isolated analyses. Recordings were analyzed by one trained aEEG analyst, who was blinded to infant factors. All aEEG data were used in context of the infant’s EGA (due to infants being within 3 days of birth), as PMA is related to maturity of the aEEG tracings.

### Neurobehavioral outcome

Between 34–42 weeks PMA, infants were assessed with the NICU Network Neurobehavioral Scale (NNNS) and the Hammersmith Neonatal Neurological Evaluation (HNNE). The NNNS is a 115-item comprehensive assessment that takes approximately 20–25 min to assess and provides summary scores of orientation, arousal, self-regulation, stress, excitability, lethargy, handling, hypertonia, hypotonia, suboptimal reflexes, quality of movement, and asymmetry. The habituation items were not scored for this study, due to the need for a quiet environment, which could not consistently be achieved in the NICU setting. The NNNS has been used extensively in research of preterm infants [27], and scores prior to NICU discharge relate to neurodevelopmental outcomes [28]. The HNNE is a 34-item assessment that takes approximately 10–15 min to complete. We used the total HNNE score as an outcome, which ranges from 0–34. The HNNE has also been used extensively in neonatal research and has been related to later outcomes [29, 30]. Due to the relationship of PMA to early neurobehavior [31], we have also captured and controlled for PMA at the time of testing for all analyses investigating relationships with neurobehavior and feeding at term.

### Feeding outcome

Feeding outcome was defined by performance on the Neonatal Oral Motor Assessment Scale (NOMAS) between 34–42 weeks PMA, as the infant was approaching discharge. This 28-item observation of jaw and tongue movements during the first 2 min of oral feeding defines if the infant is normal, disorganized, or dysfunctional. Scores have been related to long-term feeding outcomes [32, 33]. We also captured the PMA at the time of feeding assessment.

### Developmental outcomes at school age

When infants were between 5–7 years, parents were sent a questionnaire to complete. In the questionnaire there were the following assessments: Sensory Profile Short Form, Pediatric Eating Assessment Tool (Pedi-EAT), the Behavioral Pediatric Feeding Assessment Scale (BPFAS), the Behavior Rating Inventory for Executive Function (BRIEF), and the Receptive Language Subdomain of the Vineland Adaptive Behavior Scales (VABS). The Sensory Profile assesses sensory processing, is used in clinical practice and research, and has good test-retest reliability (*α*  =  0.81–0.90), validity, and internal consistency (*α*  =  0.83) [34]. The PediEAT is a parent-report assessment that measures symptoms of feeding problems in young children aged 6 months to 7 years and has excellent internal consistency, good to excellent test-retest reliability, and established construct validity [35, 36]. We also used another measure of feeding outcome that is well-described in the literature, the BPFAS. The BRIEF is a family of rating scales that were developed to capture behavioral manifestations of executive dysfunction from age 2–90 [37]. The BRIEF has strong internal consistency and high test-retest reliability [37]. Due to the population being close to school entry and many questions on the BRIEF related to organization of school materials and doing homework, an option of ‘not applicable’ was part of the questionnaire. When parents answered, ‘not applicable’, it was coded as ‘sometimes observed’ in line with how missing items are scored; however, no limitation on the number of recoded variables was employed. T-scores for the BRIEF were used as outcomes. The VABS is a standardized assessment tool that utilizes semi-structured interview or survey to measure adaptive behavior and support the diagnosis of intellectual and developmental disabilities, autism, and developmental delays [38]. The VABS-II has demonstrated moderate to high reliability in terms of subdomain scores, with the receptive communication domain having split-half reliability coefficients ranging from 0.78–0.80 for children aged 5–7 years [39, 40].

### Death or disability

To define death or disability, one composite score was defined from all of the outcome assessments at age 5–7 years, based on how each respective tool defines developmental challenges or disability. This was defined as death or any of the following: any Sensory Profile score defined as ‘probable difference’ or ‘definite difference’, a PediEat score > 95% indicating ‘high concern’, BRIEF T-score > 65 indicating ‘clinically elevated’, VABS receptive language subscale v-score identified as ‘low’ or ‘moderately low’, or BPFAS total frequency score > 84 indicating significantly higher than the normative mean.

### Statistical analysis

Statistical analyses were conducted using IBM SPSS (version 28). All medical, sociodemographic, and environmental factors were investigated for associations with total Burdjalov cerebral maturation scores in the first 72 h, corrected for EGA. All those factors that were related to the aEEG scores (when *p* < 0.05) were further considered for inclusion in a multivariable regression model to investigate relationships of aEEG to outcomes. When variables that were considered for inclusion in the multivariable regression model were co-linear, one variable was selected to include, with rationale given. Relationships between Burdjalov cerebral maturation scores, corrected for EGA, and neurobehavioral and feeding outcomes in the NICU (corrected for PMA at the time of testing), as well as developmental outcomes at ages 5–7 years, were investigated using linear regression models for continuous outcomes and logistic regression models for categorical outcomes. A multivariable linear or logistic regression model was then used to determine the relationships of aEEG scores (corrected for EGA) and neonatal (corrected for PMA) and school-age outcomes while controlling for other medical and sociodemographic factors related to early aEEG. Finally, due to the relationships of socioeconomic status and outcomes, we re-ran all analyses additionally controlling for insurance type, as a proxy for socioeconomic status.

## Results

Among the consecutive admissions recruited during the study time periods who met inclusion criteria, 64 were enrolled and received aEEG in the first 72 h. Nine (14%) expired prior to NICU discharge. Forty-four (80%) had outcome assessments at term age prior to NICU discharge. Reasons for not receiving an assessment included not being medically stable enough to participate or lack of availability of an assessor. Self-report measures were sent out to parents of all 55 remaining infants in the study at 5–7 years, and we are unaware of any infants who expired after discharge. Thirty (55%) parents completed the questionnaire of self-report measures on outcomes at age 5–7 years. Reasons for lack of participation included parent choice or inability to locate the parent/unknown contact information. See Table 1 for characteristics of the sample and relationships to aEEG.

**Table 1:** Sample characteristics and relationships to aEEG total Burdjalov score.

Categorical variables (*n* = 64)	*n*	Percentage	*p*-value^a^
Maternal factors			
Maternal marital status, married	27	42%	**0.02**
Maternal prenatal smoking	13	20%	0.29
Maternal prenatal use of alcohol	0	0%	^b^
Maternal prenatal illicit drug use	4	6%	0.50
Maternal education, Some college (*n* = 62)	28	45%	0.34
Prenatal steroids	48	75%	0.41
Delivery, Cesarean	37	58%	**0.04**
Prenatal magnesium	37	58%	0.29
Prenatal Indomethacin	0	0%	^b^
Maternal spinal anesthesia	36	56%	0.49
Maternal general anesthesia	13	20%	0.61
No spinal or general anesthesia	15	23%	0.59
Labor antibiotics	30	47%	0.41
Infant factors			
Race (African-American/Black)	34	53%	**<0.01**
Insurance type, public (*n* = 46)	28	61%	0.66
Infant sex, female	25	39%	0.84
Cerebral injury (IVH grade III or IV or cystic PVL)	25	39%	**0.04**
PDA (*n* = 63)	44	70%	0.90
NEC (*n* = 61)	12	20%	0.32
ROP (*n* = 61)	13	21%	0.55
Expired	9	14%	0.69
Environment			
NICU room type, single patient room	30	47%	0.61
Concurrent endotracheal intubation	45	70%	0.56
Concurrent use of sedatives	24	38%	0.34

Continuous variables	Mean or median	Standard deviation or interquartile range	*p*-value^a^
Maternal factors			
Maternal age, yrs	26.9	5.6	0.31
Number of prenatal visits (*n* = 63)	4.1	2.3	0.46
Infant factors			
*EGA, wks	25.5	1.35	**0.02**
Birth weight, grams	830.6	191.8	0.17
Occipital-frontal circumference at birth, cm	23.2	1.7	0.81
Apgar, 1 min	3.5	2.3	0.84
Apgar, 5 min	5.8	1.8	0.59
CRIB II score	11	(10–13)	0.29
Cord gases (n = 40)	7.32	0.08	0.63
Number of days on TPN, days	18	(11.25–33)	0.89
Number of days on HFOV, days	0	(0–5)	0.34
Number of days with endotracheal intubation, days	9	(1–34)	0.056
Number of days on CPAP, days	13	(4–35)	0.23
Number of days on nasal cannula, days	19	(7.5–30)	0.92
Total oxygen hours, hrs	2256	(1380–2760)	0.85
Total number of days on breast milk, days	40	(26–74)	0.91
Length of stay, days	104	(76–126.5)	0.97

*IVH* intraventricular hemorrhage, *PDA* patent ductus arteriosus (requiring Indomethacin or surgical ligation), *NEC* necrotizing enterocolitis (Bell’s stage IIb or higher), *ROP* retinopathy of prematurity (requiring surgery or Bevacizumab treatment), *EGA* estimated gestational age, *CRIB-II* Clinical Risk Index for Babies II, *TPN* total parenteral nutrition, *HFOV* high frequency oscillatory ventilation, CPAP continuous positive airway pressure.

^a^*P*-value is from investigating the relationships of each factor with total Burdjalov cerebral maturation score, while controlling for EGA using linear regression analyses. Bolded values indicate significance (*p* < 0.05).

^b^Unable to calculate due to variable being a constant.

^*^The relationship between EGA and total Burdjalov cerebral maturation score was assessed using a univariate model.

The aEEG Burdjalov score timeframe for midpoint analysis ranged from 25–63 h of life with a mean (SD) of 47.3 (8.4) hours. See Table 2 for descriptives of the aEEG scores of the sample.

**Table 2:** aEEG cerebral maturation scores from the sample.

aEEG Burdjalov cerebral maturation scores (*n* = 64)	Median (IQR)
Total score	3.0 (2.0–5.8)
Continuity	1.0 (0.0–1.0)
Cycling	0.0 (0.0–1.0)
Amplitude of lower border	1.0 (1.0–2.0)
Bandwidth span	1.0 (1.0–2.0)

### Relationships between aEEG and infant medical, maternal, and environmental factors

EGA was related to total Burdjalov cerebral maturation scores (*p* = 0.02). Medical factors that were related to total Burdjalov cerebral maturation scores, corrected for EGA, included: type of delivery (*p* = 0.04), infant race (*p* < 0.01), marital status (*p* = 0.02), and cerebral injury (*p* = 0.04). Relationships between Burdjalov cerebral maturation scores and number of days of endotracheal intubation failed to reach significance (*p* = 0.056). There were no other relationships between Burdjalov cerebral maturation scores and medical, social, or environmental factors.

Although EGA, type of delivery, infant race, marital status, and cerebral injury were all considered for inclusion in the multivariable regression model due to their relationship with aEEG scores, several variables were colinear. Therefore, further selection of appropriate variables to go in the multivariable model was warranted. Race and marital status were related (*p* < 0.001), which could be expected based on past research [41]. Therefore, a combined variable indicating if the infant was either Black race or from a single parent was included in the multivariable model in an effort to include both factors. Brain injury and EGA were related to each other (*p* = 0.04), so only EGA was maintained in the model as a critical component related to maturation of the Burdjalov score. Therefore, the multivariable model controlled for EGA, delivery type, and a combined variable representing race and marital status. Analyses of outcomes at term age also controlled for PMA at the time of testing.

### Relationships between aEEG and neurobehavioral and feeding outcomes

See Table 3 for relationships between Burdjalov scores and neurobehavioral and feeding outcomes during NICU hospitalization as well as relationships between Burdjalov scores and developmental outcomes at 5–7 years of age. After re-running all analyses additionally controlling for insurance type, as a proxy for socioeconomic status, the significant findings remained unchanged.

**Table 3:** Relationships of total Burdjalov cerebral maturation scores, corrected for EGA, and outcomes.

	Test statistic (t)	Standardized beta	^a^*p*-value	^b^Multivariable *p*-value
Infant outcome at term				
Death prior to NICU discharge		0.02	0.89	
HNNE	0.44	0.09	0.66	
NNNS Orientation	0.59	0.13	0.56	
NNNS Handling	1.31	0.26	0.20	
NNNS Quality of Movement	−1.27	−0.24	0.21	
NNNS Self-Regulation	0.05	0.01	0.96	
NNNS Non-optimal Reflexes	−1.73	−0.34	0.09	
NNNS Stress	−186	−0.34	0.07	
NNNS Arousal	2.38	0.48	**0.03**	**0.02**
NNNS Hypertonicity	0.69	0.13	0.10	
NNNS Hypotonicity	−1.80	−0.35	0.08	
NNNS Asymmetric Reflexes	−0.62	−0.13	0.54	
NNNS Excitability	1.31	0.29	0.14	
NNNS Lethargy	−1.34	−0.26	0.19	
NOMAS Feeding		0.44	0.16	
**Childhood outcomes at 5–7 years**				
BRIEF Inhibit	−2.98	−0.60	**0.02**	**0.007**
BRIEF Shift	−1.96	−0.39	0.06	
BRIEF Emotional Control	−1.63	−0.35	0.12	
BRIEF Initiate	−1.78	−0.39	0.09	
BRIEF Working Memory	−2.61	−0.55	**0.007**	**0.02**
BRIEF Plan/Organize	−1.81	−0.37	0.08	
BRIEF Organization of Materials	−2.92	−0.58	**0.00**2	**0.008**
BRIEF Monitor	−1.96	−0.39	0.06	
BRIEF Metacognitive	−2.85	−0.58	**0.01**	**0.01**
BRIEF Behavioral Regulation	−2.49	−0.51	**0.03**	**0.02**
BRIEF Global Executive	−3.17	−0.62	**0.002**	**0.004**
Sensory Profile Tactile Sensitivity	1.25	0.27	0.22	
Sensory Profile Taste/Smell Sensitivity	0.29	0.06	0.77	
Sensory Profile Movement Sensitivity	−1.77	−0.35	0.09	
Sensory Profile: Under-responsive/Seeks Sensation	0.94	0.19	0.35	
Sensory Profile: Auditory Filtering	1.43	0.29	0.16	
Sensory Profile: Low Energy/Weak	0.64	0.13	0.53	
Sensory Profile: Visual/Auditory Sensitivity	1.81	0.34	0.08	
Pedi-EAT	−0.91	−0.19	0.38	
BPFAS	−1.43	−0.29	0.17	
VABS - Receptive Language	1.04	0.21	0.31	
Death or disability	0.22	0.04	0.83	

Outcomes at term age also control for PMA at the time of testing. Categorical outcomes (death in NICU, NOMAS feeding, and death or disability) investigated using multivariable logistic regression models. Death or disability was defined from all of the outcome assessments at age 5–7 years, based on how each respective tool defines developmental challenges or disability. This was defined as death or any of the following: Any Sensory Profile score defined as ‘probable difference or ‘definite difference’, Pedi-Eat score > 95% indicating ‘high concern’, BRIEF t score > 65 indicating ‘clinically elevated’, VABS receptive language subscale v-score identified as ‘low’ or ‘moderately low’, or BPFAS total frequency score > 84 indicating significantly higher than normative mean. Bolded values are significant (*p* < 0.05).

*NNNS* NICU Network Neurobehavioral Scale, *HNNE* Hammersmith Neonatal Neurological Evaluation, *NOMAS* Neonatal Oral Motor Assessment Scale, *Pedi-EAT* Pediatric Eating Assessment Tool, *BRIEF* Behavior Rating Inventory for Executive Function, *BPFAS* Behavioral Pediatric Feeding Assessment Scale, *VABS* Vineland Adaptive Behavior Scales.

^a^*P*-value is from investigating relationships between Burdjalov total scores in the first 3 days of life and outcome at term and 5–7 years using linear regression models while controlling for EGA.

^b^Multivariable *p*-value is from multivariable linear regression controlling for EGA, PMA at the time of testing (for outcomes at term age), in addition to delivery type and a combined variable representing race and parent marital status.

## Discussion

In this study, vaginal birth as well as adverse medical factors such as cerebral injury were associated with worse aEEG markers in the first 72 h of life for extremely preterm infants. Lower aEEG scores were also noted among infants who were born to unmarried parents and who were Black. Lower Burdjalov cerebral maturation scores on early aEEG within the first 72 h after extremely preterm birth were also related to poorer arousal on neurobehavioral assessment at term and executive dysfunction at 5–7 years of age. We did not observe relationships of early aEEG to feeding outcomes or sensory processing measures.

The first 72 h after extremely preterm birth is a critical period where the infant must undergo significant physiological adaptation to cope with changes in the environment. Failed or inadequate compensation is associated with increased risk of cerebral injury [42] and is reflected in functional measures such as the aEEG Burdjalov cerebral maturation score, with poorer scores among infants with cerebral injury having been previously defined [18–20] as well as identified in the current study. However, the directionality of this relationship is unclear; such alterations in brain activity on aEEG may be a signal of existing cerebral injury or may be a marker that injury is about to occur. Either direction would be important clinical information, yet with different implications.

Poorer aEEG scores were observed among preterm infants who had a vaginal delivery. A previous systematic review and meta-analysis identified higher risk of death following vaginal delivery among extremely low birth weight infants, but did not appreciate an increased risk of intraventricular hemorrhage [43]. However, there is also mixed evidence on type of delivery with some studies identifying no differences in brain outcomes or mortality based on delivery mode [43–45], and still others observing decreased risk of mortality and lower rates of intraventricular hemorrhage with Cesarean delivery in very low birth weight infants [46–48]. Although our study demonstrated relationships between vaginal delivery and lower Burdjalov scores, causal relationships cannot be established from this observational study. Further, there has been increased interest in use of aEEG during the perinatal period to assess cerebral activity during the transition from the intrauterine to the extrauterine environment [49]. The use of such monitoring of electrocortical activity may provide useful and potentially more discriminative insights to aid our understanding of concurrent brain activity that puts the infant at a higher risk of subsequent alterations [50, 51]. This could improve our understanding of concurrent effects on the brain, compared to other studies that may be limited by the sensitivity of brain injury measures and the longitudinal nature of waiting for neurodevelopmental outcomes to unfold.

Unexpectedly, poorer Burdjalov scores were also associated with sociodemographic factors including having unmarried parents and Black race. Although there is a high percentage of children born to unmarried parents in the study sample [52], there is colinearity between the variables of marital status and race. While there is an increasing recognition of worse outcomes for Black very low birth weight infants, including an increased risk of cerebral palsy [53] and death [54], there has been relatively limited study of potential contributions due to differences within the NICU environment itself. The poorer Burdjalov scores identified in this study may reflect a greater level of medical illness in these infants or may reflect toxic stress [55] during pregnancy, as brain immaturity has been previously associated with poverty and pregnancy stress [56, 57]. It will be important in future studies to prospectively capture measures of race/ethnicity, poverty, and detailed maternal health parameters to better disentangle the potential roles of health disparities, medical illness, and the impact of care practices on infant health, while identifying differences in functional maturation and outcomes.

Burdjalov scores shortly after birth were related to poorer arousal at term age, just prior to NICU discharge, which occurs on average approximately 3 months after extremely preterm birth (median length of stay was 104 days). There are no other studies, that we are aware of, that have investigated the relationship between early aEEG within the first 72 h and neurobehavior at term. However, aEEG at 30 weeks PMA has been related to motor outcomes at term equivalent age [12]. Burdjalov scores in this sample were not related to death prior to discharge, which is inconsistent with other research that has demonstrated both Burdjalov and Hellstrom-Westas scoring systems for cyclicity as being related to survival [17]. It is also possible that the later PMA of EEG recording in that study led to a biased study population (those that survived the first week of life, when mortality is greatest for very low birthweight infants).

EEG measures have been used to identify differences in children with sensory processing disorders [58, 59]. However, we are not aware of studies that have investigated the risk of sensory processing problems or alterations to the development of sensory processing through the use of aEEG. In this study, early Burdjalov scores were not related to any of the sensory processing scores obtained at ages 5–7 years. Early functional differences would be anticipated to relate to foundational differences in sensory-motor development, especially given the aEEG electrode placement over the motor strip. However, the aEEG does not reflect all of the same factors as EEG, such as rhythm and frequency, which could add to discriminative capability. Further, the time compressed nature of aEEG may limit visualizations of dynamic and functional changes. It is also possible that the sensory processing measure that was used is not sensitive enough to detect subtle differences in sensory processing or that there was too much time between the aEEG measure and sensory processing outcome to account for other influences. We also were not able to demonstrate a relationship between aEEG and childhood feeding.

This is one of only a few studies to investigate measures of executive functioning in extremely preterm infants, with the current study having outcomes at ages 5–7 years in relation to aEEG cerebral maturation scores taken within the first 72 h of life. Executive functioning includes complex cognitive processes that allow us to plan, organize, implement, evaluate, and restructure our behaviors. It includes cognitive skills such as working memory, flexible thinking, and self-control. Executive functioning arises predominantly from the prefrontal cortex, an area of the brain not covered well by conventional C3-P3, C4-P4 electrode placement in typical aEEG recording. However, injury (including white matter injury [60]) is associated with decreased executive functioning at preschool age, highlighting the importance of connectivity throughout the brain to support complex cognitive and motor tasks. In this context, it is interesting, but not surprising to see that such aEEG Burdjalov scores, as a global marker of cerebral function, were related to multiple areas of executive function under the metacognitive and behavioral regulation indexes, including inhibition, working memory, and organization of materials. These all require coordinated activity throughout the brain. Our findings are consistent with other studies that have identified aEEG or EEG to relate to cognitive outcomes at school age [22–24].

This study has several limitations. First, it is an observational study with a small sample size that was part of an overarching study, meaning it was not powered for these analyses. Future investigations can use larger samples defined by a power analysis. This study used aEEG in the first 72 h of life, a time in which significant artifact can be introduced due to the complex medical environment, reducing the total time available for optimal analysis of the aEEG tracing. The EGA may not have been precise, which could have impacted the findings. Not all maternal and infant medications were documented and could have impacted aEEG scores. Other factors within the NICU environment, such as the volume of auditory exposure or other sensory exposures, were not captured in the current study and would be important areas for future inquiry. It remains unclear if the part of the tracing that was analyzed represented the infant’s function globally. This study was limited to the first 72 h of life and did not account for changes in cerebral function which may have occurred over the remainder of NICU hospitalization. Future investigation may benefit from serial assessments across time. Further, the timing of cranial ultrasounds to screen for cerebral injury and term-equivalent MRI necessarily means that the exact timing of brain injury (inclusive of IVH, white matter injury, and cerebellar hemorrhage) cannot be determined with a high degree of certainty. Thus, the directionality of aEEG changes and injury cannot be assessed. This study used parent-report measures of outcome at 5–7 years of age, which can yield different information than observational evaluation. Specifically, poor relationships between observational and parent report measures, such as the BRIEF, have been reported [61, 62]. This study also is unable to fully capture nor disentangle the significant number of medical, sociodemographic, and environmental factors that can impact both aEEG as well as outcomes. It also did not capture or classify other developmental challenges that could better represent the child’s outcomes. In addition, not all infants could be assessed at term nor did all families complete the self-report measures at 5–7 year follow-up, which could introduce bias. Finally, this investigation relied on multiple statistical analyses across many outcome variables, which could have increased the risk of a Type I error.

The use of aEEG in the neonatal intensive care unit continues to expand. It provides a relatively low-cost bedside evaluation of cerebral health that can be interpreted by trained personnel. Although the primary clinical focus of aEEG use is real-time monitoring for seizures and brain function, the accessibility of this modality lends itself well to investigations of other markers of short- and long-term brain health. The results of this study confirm the findings of others that aEEG markers can indicate concurrent medical conditions, such as cerebral injury [16], and also provides insight into novel use, demonstrating a link between early dysfunction and executive function deficits at school age (5–7 years).

Further research to expand this preliminary work should aim to better define the trajectory of cerebral maturation scores over the entire NICU course and assess the relationship with later functional outcomes. An improved understanding of this connection will help to select and implement targeted interventions with the aim of improving outcomes for infants at greater risk.

## Data Availability

The datasets generated during and analyzed during the current study are available from the corresponding author on reasonable request.
